# Understanding AI and power: situated perspectives from Global North and South practitioners

**DOI:** 10.1007/s00146-025-02731-x

**Published:** 2025-11-14

**Authors:** Venetia Brown, Retno Larasati, Joseph Kwarteng, Tracie Farrell

**Affiliations:** Knowledge Media Institute, The Open University, Milton Keynes, United Kingdom

**Keywords:** Artificial intelligence, AI narratives, AI harms, AI ethics, Power asymmetries

## Abstract

Global debates on artificial intelligence (AI) ethics and governance remain dominated by high-income, AI-intensive nations, marginalizing perspectives from low- and middle-income countries and minoritized practitioners. This qualitative study adopts a decolonial and sociotechnical lens to examine how AI practitioners across Africa, Asia, South America, the Caribbean, and minoritized groups working in high-income contexts conceptualize AI’s value, harms, and governance. Drawing on reflexive thematic analysis of 22 in-depth interviews, the study explores how geographic, cultural, and professional contexts shape practitioners’ understandings of ethics, harm, and power within the global AI ecosystem. Findings reveal a dual orientation. While some participants view AI as a neutral tool shaped by human intent, others frame it as a sociotechnical system that reproduces structural inequities through data colonialism, exclusion, and epistemic dependency. Despite these asymmetries, participants articulated cautious yet agentic imaginaries of AI’s potential to address local and regional problems in healthcare, education, and public governance. The study advances decolonial AI ethics by empirically grounding how ethical reasoning and governance are negotiated under constraint and by highlighting pathways toward more equitable, context-sensitive global AI governance.

**Supplementary Information:**

The online version contains supplementary material available at 10.1007/s00146-025-02731-x.

## Introduction

Artificial intelligence (AI) is widely regarded as a transformative technology that reshapes societies and impacts multiple sectors, offering significant benefits but also posing uneven risks, particularly for countries in the Global South (GS) and vulnerable populations. Despite growing attention to AI ethics, the majority of scholarship continues to reflect Western, Eurocentric perspectives, leaving situated accounts from low- and middle-income countries (LMICs) underexplored (Barrett et al. 2025; Okolo 2021). This imbalance reflects entrenched structural asymmetries in authority and agenda setting. Institutions in the Global North (GN) largely determine the strategic priorities, financial resources, and technological narratives that shape AI development and governance. These narratives not only reflect particular imaginaries for society but also reproduce long-standing social, economic, and political inequalities (Sartori and Theodorou 2022), thereby influencing which values and risks are prioritized and whose perspectives are amplified or marginalized.

These enduring power asymmetries are not only institutional, reflected in the financial and strategic dominance of GN actors, but also epistemic, determining whose expertise and perspectives are rendered authoritative. Structural barriers in research infrastructure and epistemic authority limit the visibility and legitimacy of certain voices in AI research (Safir et al. 2025). In local contexts, researchers from LMICs are often confined to narrowly technical or implementation roles, rather than contributors to broader ethical or governance debates (Brown et al. 2024). These exclusions persist even when mobility occurs,as language hierarchies in academic publishing and migration to wealthier regions, often driven by opportunities within large technology firms, further diminish the visibility of locally grounded expertise. This challenge is compounded by what Leonard (2023) describes as the technology industry’s entrenched cultures of discrimination and exclusion, which reproduce systemic barriers even within ostensibly global research spaces. Collectively, these dynamics reinforce epistemic asymmetries and constrain the surfacing of perspectives grounded in diverse sociotechnical realities

A review of qualitative research on AI’s societal impacts (2013–2023) found that most studies involved experts from North America and Europe, with perspectives from LMICs largely absent (Brown et al. 2024). This imbalance is problematic, as those most vulnerable to AI-related harms are often least represented in influencing discourse, leaving their lived experiences, priorities and contextual knowledges largely overlooked.

Similar patterns emerge in ethical frameworks and principles that disproportionately reflect GN priorities. Although these frameworks have advanced governance debates, they abstract ethical reasoning from the political and material conditions that shape harm and sideline alternative perspectives (Jobin et al. 2019). Consequently, decolonial scholars have critiqued the universality of Western moral reasoning and Eurocentric ethical principles and frameworks, instead advocating for more pluralistic and culturally grounded approaches that reflect diverse epistemologies and local contexts (Birhane 2021; Mhlambi 2020; Okolo, 2024).

Building on calls to decolonize AI ethics and center underrepresented voices in AI research, this study examines how geographic, cultural, and professional contexts shape AI practitioners’ understanding of harm, ethics, power, and AI value. Drawing on 22 in-depth qualitative interviews, we foreground practitioners (i.e., professionals engaged in AI research, development, governance or education) as knowledge producers whose perspectives are situated within historically contingent sociotechnical systems. We examine how their situated viewpoints engage with global structural asymmetries in AI governance, providing insights from voices often marginalized in dominant ethics discourse. Our sample spans five continents and includes participants from low- and middle-income countries (LMICs), upper-middle-income countries (UMICs), as well as minoritized practitioners working within high-income countries (HICs), offering a pluralistic lens on ethics and the sociotechnical implications of AI.

To address this gap, we pose four research questions across distinct dimensions. **RQ1** explores how practitioners conceptualize AI and harm, engaging assumptions underpinning dominant ethics discourse. **RQ2** examines how they navigate the fluid and contested meanings of ethical AI. **RQ3** investigates how practitioners experience and interpret power asymmetries within the global AI ecosystem. Finally, **RQ4** explores how practitioners perceive AI’s value in addressing societal challenges, highlighting tensions between aspirational narratives and context-specific realities.

The remainder of this paper is organized as follows. Section 2  reviews literature on AI ethics frameworks, contrasting dominant paradigms with decolonial-informed critiques to establish the conceptual foundations of our study. Section 4 outlines our methodological approach and the cross-regional interviews conducted with AI practitioners. Section 4.3.2 presents our findings across four themes, reporting practitioners’ perspectives around harm, ethics, power, and AI’s values. Section 4.4 discusses these insights in relation to broader debates on global AI governance and epistemic inequality. The paper concludes in Section 6, followed by  Section 7, which reflects on implications and future research for more inclusive approaches to AI ethics.

## Background

For clarity, we use Global North (GN), Global South (GS), low- and middle-income countries (LMICs), upper-middle-income countries (UMICs), and high-income countries (HICs) as established in the Introduction. These categories are structural, rather than geographical, descriptors of asymmetries in power, resource and epistemic authority in the global AI ecosystem. We use GS to foreground historical marginalization and inequality, especially in LMICs/UMICs, acknowledging the term’s evolving and contested nature (Arun 2020). The categories broadly follow The World Bank^1^ income grouping (The World Bank Group, 2025), while we recognize that such typologies simplify complex histories and politics. In the following sub-sections, we trace how ideas about AI shape ethics in practice, how power consolidates procedural norms towards relational and decolonial re-framings, and how this cultivates in a focus on harm and values.

### AI determinism and sociotechnical framing

Debates on AI’s development reveal divergent assumptions about the relationship between technology and society. Research often emphasizes computational artifacts aimed at automation and augmentation, typically framed through deterministic views that portray AI as an autonomous engine of process—predictable, linear, inevitable and socially transformative—promising social benefits through innovation and efficiency (Johnson and Verdicchio 2024). This framing, prevalent in national agendas, policy documents, and industry discourse, presents technological advancement as neutral and universal while concealing the geopolitical and material infrastructures that enable it (Birhane 2021; Mohamed et al. 2020). Such imaginaries privilege Western models of progress and obscure the uneven distribution of labor, data, and environmental costs across the AI global supply chain.

In contrast, Science and Technology Studies (STS), decolonial and political theory scholarship perspectives (Mhlambi and Tiribelli 2023; Rafanelli 2022; Ricaurte et al. 2024) challenge this teleological view by emphasizing the contingent and co-produced nature of technology. From this perspective, AI systems are not autonomous artifacts with fixed properties but sociotechnical assemblages mediated by dynamic interactions and complex interplays of social, cultural, technical, and political economies (Johnson & Verdicchio 2024). This view rejects technological neutrality, highlighting that algorithms embed values and reproduce systemic inequities, including racialized and capitalist logics (Katz 2020; Tsamados et al. 2021). The tension between these perspectives highlights that what is often framed as technological progress is, in practice, the outcome of situated human choices and global power relations. Building on this insight, a sociotechnical framing challenges dominant narratives of AI innovation and re-situates it within the social, political, and economic asymmetries that structure its production. It, therefore, opens space to interrogate how global asymmetries shape whose ethics, values, and futures are made visible in AI development. Understanding AI in these terms reframes ethics as a situated rather than universal practice. Whereas GN approaches emphasize fairness and transparency, decolonial traditions foreground relational ethics, data sovereignty and collective responsibility—tensions explored in the next section.

### Global North logics of AI governance

The ethical governance of AI has been largely articulated through frameworks originating in the GN (Jobin et al. 2019). Early discussions centered on technical performance metrics, but ethical principles such as fairness, accountability, and transparency now anchor the vocabulary of responsible AI (Floridi et al. 2018). These principles reflect a procedural orientation—an ethics of process and measurement that favors quantifiable accountability and bias mitigation over questions of social and distributive justice. Despite this broader scope, the field remains fragmented, with competing frameworks continuing to reflect GN priorities and epistemologies. This tension is evident in global standard setting, where cooperation flourishes on technical specifications but falters on ethical governance (von Ingersleben-Seip 2023). The ease of consensus on the technical, and the difficulty of agreement on the ethical, reveal how dominant frameworks value procedural measurability over plural and relational understandings of responsibility.

A similar pattern appears in regulatory governance. The European Union’s AI framework—often described through the “Brussels effect”—illustrates how fairness, accountability, and transparency are codified as global norms (Dempsey et al. 2024). Although such initiatives strengthen accountability and rights protection, their diffusion also universalizes a distinct European conception of ethics. In doing so, GN regulatory power consolidates procedural logics of governance while constraining relational, context-specific approaches.

These dynamics extend beyond regulation into the wider architecture of AI governance. Reviews show that most ethical guidelines originate in the GN, particularly Western Europe and North America, and are influenced by government and corporate interests (Corrêa et al. 2023; Gornet et al. 2024). Framed as universal, these documents project Western epistemologies of ethics and expertise while marginalizing perspectives from regions most affected by AI’s impact. Critics argue that the guidelines remain overly abstract, lacking actionable mechanisms and attention to structural inequalities or historical responsibility (Birhane 2021; Whittlestone et al. 2019). The result is a proliferation of principles that signal virtue but offer limited traction on questions of justice or inclusion.

Similar structural tendencies are evident in research practice. AI researchers, particularly in AI-intensive nations, tend to prioritize technical fairness and bias mitigation while giving less attention to broader social and distributive impacts (Pant et al. 2024; Sartori and Theodorou 2022). Johnson (2024) attributes this fragmentation to fluctuating definitions and standards, while Frank and Klincewicz (2024) note that AI systems inherently embody values, urging discernment between genuine moral concerns and a “firehouse of fears, misunderstandings and competing narratives” (p.205). Although researchers from LMICs are increasingly recruited by prestigious institutions in HICs, their participation seldom translates into epistemic influence, as dominant agendas continue to prioritize the GN. Consequently, ethical reflection risks becoming a technical exercise rather than a social or political one, reinforcing the procedural logic that define GN governance (Ali et al. 2024).

### Relational and decolonial logics of AI ethics

Extending critiques of the procedural logics that dominate GN frameworks, relational and decolonial perspectives reframe ethics as a situated and social practice. Rather than treating ethical compliance as a checklist or technical fix, relational ethics center reciprocity, care, and community well-being, recognizing that moral responsibility is constituted through relationship rather than mere abstraction. Decolonial perspectives interrogate the epistemic hierarchies that define whose knowledge and values guide AI design and governance. In their different ways, these perspectives return ethics to the terrain of lived experiences and collective responsibility, reminding us that questions of technology are always questions of relation.

There have been growing calls for more inclusive and context-sensitive ethical frameworks that recognize diverse cultural, historical, and political realities. Mhlambi (2020) draws on the *Ubu-Ntu* philosophy to propose a relational understanding of personhood grounded in community and shared humanity rather than individual autonomy. Birhane (2021) challenges rationalist paradigms by arguing that ethics should center lived experiences of those most impacted by algorithmic systems. Racine (2024) and Barrett et al. (2025) offer justice-oriented frameworks that combines culturally grounded participatory and intersectional principles, centering agency, communal values, and historical awareness. Across these interventions, ethics is addressed through plurality, situatedness, and repair and considers the moral reflection of who is seen, heard, and empowered within technological development.

Decolonial ethics deepens this relational orientation by confronting the structural and historical forces that shape global AI governance. The call for decolonial ethics arises from long-standing patterns of extractive practices and power consolidation of decision-making authority between the GN and GS (Belli and Gaspar 2024). The infrastructures and data economies underpinning AI often replicate earlier colonial patterns of resource and labor extraction, where regions in the GS provide data, annotation work, and computational resources while deriving limited benefit (Mejias and Couldry 2024; Quijano 2000). These continuities reveal how AI development extends a broader trajectory of technological dependency and exploitation, where global hierarchies of value and expertise remain deeply entrenched. Against this backdrop, decolonial ethics call for frameworks that address both material and epistemic injustice, making visible the histories that technological narratives often obscure. Table 1 summarizes the procedural and relational logics that characterize GN and GS approaches to AI governance and ethics, as discussed in this section.

**Table 1:** Contrasting Global North and Global South approaches to AI ethics and governance

	Global North approaches (procedural logics)	Global South/decolonial approaches (relational logics)
Ethical orientation	Emphasizes technical process and measurement; ethics framed around fairness, accountability, and transparency.	Treats ethics as contextual and ongoing; grounded in lived experience, care, reciprocity, and collective responsibility.
Scope of governance	Focuses on bias mitigation, explainability, accountability within existing institutional systems.	Seeks to address structural inequality and historical marginalization in AI development and use.
Knowledge and expertise	Privileges institutional and technical expertise from high-income regions.	Values situated, plural knowledges including Indigenous and community-based perspectives.
Common critiques	Overly abstract, inattentive to inequality; ethics reduced to checklists or symbolic principles.	Sometimes fragmented or under-resourced but foregrounds justice, repair, and collective well-being.
Underlying view of justice	Procedural justice—fairness through rules and transparency.	Relational or restorative justice—repair, reciprocity, and shared accountability.

### Conceptions of AI harm and global inequalities

Discourses of harm occupy a central but uneven place in the governance and imagination of AI. Within dominant GN narratives, harm is typically understood as a procedural or technical failure (e.g., as bias, inaccuracy, opacity) amendable to mitigation through fairness metrics or explainability tools. Such framings narrow the moral field to questions of model performance while obscuring the wider, sociopolitical and ecological conditions that make harm possible (Franzke 2022). From a sociotechnical perspective, however, harm is not an error within neutral systems but a symptom of how power, labor, and data are unevenly organized and distributed across global infrastructures. What counts as harm, and who is recognized as harmed, thus reflects existing hierarchies of visibility, expertise, and value in the AI ecosystem.

AI systems and technologies frequently reproduce and intensify these asymmetries. Scholars identify material harms in the extractive use of energy and natural resources required to sustain AI infrastructures (Anson et al. 2022; Mwema and Birhane 2024). Social harms manifest in algorithmic discrimination against marginalized groups and biased risk-assessment systems (Marda and Narayan 2020; Ngamita 2024). Labor harms arise through the precarious conditions of data workers and content moderators wherein low-paid labor underpins global data economies (Aguilar et al. 2021; Miceli and Posada 2022). These cases reveal the uneven effects of AI and how it functions as a sociotechnical assemblage in complex ways. Viewed through Quijano’s (2000) concept of *coloniality of power*, these patterns of extraction, inequity, and invisibility reveal how contemporary AI infrastructures reproduce historical hierarchies rooted in Eurocentric knowledge systems, racialized and social stratifications and global capital.

Despite growing awareness of these harms, a persistent belief in AI solutionism endures. This optimism assumes that complex social problems can be solved through technical means, often oversimplifying political and cultural realities (Morozov 2014, p. 5). The discourse of AI for Social Good (AI4SG) epitomizes this logic, promising innovation for humanitarian and developmental aims. Yet critics note that such initiative often reproduce the very asymmetries they claim to address (Yeung 2024). For instance, a policy brief on the Sustainable Development Goals (SDGs) found that corporate control over data infrastructure introduced new dependencies, limiting data privacy, access to digital commons and entrenchment of unequal trade and investment relations between LMICSs and the GN (O’Brien 2022). Similar concerns appear in healthcare (Turon et al. 2024) and climate research (Jolaoso 2023), where AI-driven interventions risk reinforcing rather than reducing inequality. AI4SG projects may, thus, promote benevolent uses of AI while creating the (potentially unintended) effect of reinforcing existing inequalities and asymmetrical power dynamics.

Understanding harm, therefore, requires moving beyond procedural framings to account for the structural, epistemic, and historical relations that produce vulnerabilities. The notion of harm is inseparable from the coloniality of power, underpinning global data economies and the uneven distribution of AI’s benefits and risks. Building on these insights, this study responds to this gap by examining how practitioners conceptualize harm, interpret the shifting meanings of ethical AI, navigate power asymmetries and evaluate AI’s broader societal value.

## Method

We adopted a qualitative design using semi-structured one-to-one interviews to explore how AI practitioners conceptualize harm, ethics, power, and AI’s societal value. Our approach is interpretivist and reflexive whereby we treat knowledge as situated and co-constructed between participants and researchers. The study was approved by the university’s Human Research Ethics Committee (Ref:4758) and conducted in accordance with GDPR/UK data protection requirements.

### Recruitment

Participants were recruited using purposive sampling, with limited snowballing to recruit AI practitioners who were primarily raised or educated outside Western European and North America. To capture transnational trajectories, we also included practitioners originally from low- and middle-income countries (LMICs) or upper-middle-income countries (UMICs) who currently work in high-income countries (HICs)—per World Bank income classification.

Recruitment occurred via two pathways: (1) a general call on the project’s website, and (2) direct outreach through email with project details and consent forms. We also leveraged professional networks to disseminate the call. Although our plan targeted West Africa more broadly, travel-based recruitment around conference-adjacent outreach resulted in a concentration of Ghana-based practitioners. This introduces potential network and ecosystem biases (e.g., conference homophily; shared professional norms, policy priorities specific to Ghana) that shape how harm, ethical AI, and value are articulated. We mitigate this at reporting by (i) balancing quotes across Ghana and non-Ghana participants where possible and (ii) noting stratified comparisons (Ghana vs. non-Ghana) where patterns differ. Although 8/22 participants (36.4%) were Ghanian, the dataset offers informational richness and support analytical generalizations to comparable African AI ecosystems.

Of the 22 participants, 21 were born and/or primarily educated in LMICs/UMICs. We also included one participant from an Indigenous community in North America to incorporate perspectives on AI. Participants received UK £25 honorarium, with the option to select an e-voucher aligned to their local context or ethical principles.

### Procedure

We conducted three pilot interviews with external researchers (not in the final sample and not analyzed alongside study data). Salient themes and phrasing from their answers informed the wording, probes, and sequencing of the final interview guide. The guide covered AI benefits/harms, education and information sources, current work/research activities, regional development/adoption, and future directions. Participants received information and consent forms describing the study purpose, participation requirements, confidentiality, data management and the right to withdraw. To support cultural and linguistic inclusivity, participants could request (i) a translator/interpreter and/or (ii) an interviewer from a non-Western background, should they wish.

Interviews were conducted online via Microsoft Teams and in-person (Germany, Antigua, Ghana) between March 2023 and Jan 2025, typically lasting 60–90 min. With permission, sessions were audio-recorded. The interviews followed a flexible format, encouraging discussion to unfold naturally and allowing participants to highlight issues most relevant to them. Recordings were transcribed verbatim by a certified provider and checked line-by-line by a research team member for orthography and accuracy against the audio. To further protect confidentiality and mitigate risks of deductive disclosure in small, professional communities, we implemented post-interview consent adapted from Kaiser (2009) where participants could review, restrict, or retract specific excerpts.

### Researcher positionality and reflexivity

Our research team spans GS and GN affiliations with backgrounds in HCI, AI/ML, social science, and education. These locations and professional histories shaped the language of our questions, what we recognized as salient and our sensitivity to colonial and regional power dynamics. To make this influence visible and tractable, we prepared reflexive memos from familiarization to write up and held peer-debriefs to challenge assumptions.

### Analysis

We conducted reflexive thematic analysis Bruan and Clarke (2006, 2021) to identify and interpret patterns of meaning across the dataset. Given the exploratory nature of the study and the underrepresentation of these perspectives in existing literature, we adopted an inductive approach, rather than imposing deductive themes on participants’ statements and allowing participants’ conceptualizations to emerge organically.

The six-phase RTA process was applied iteratively: familiarization → initial coding → theme development → review → definition → write-up. Transcripts and coding were managed in NVivo v14. Three researchers cross-coded a subset of interviews spanning different regions to identify overlaps and ambiguities in the preliminary code set. In line with RTA, we did not calculate inter-coder reliability. Instead, we adopted a consensus building approach (Barbour 2001) whereby the team compared coding decisions, discussed divergent interpretations, and refined code boundaries, labels and definitions through iterative dialog. These discussions distinguished semantic (i.e., explicit meaning) from latent meaning (i.e., underlying meaning) and led to a revised, shared codebook, used for subsequent coding of the remaining dataset. A coarsened demographic table and codebook are provided as online Supplementary Information.

## Findings

This section presents how AI practitioners across regions understand and navigate AI, its harms/benefits, ethical responsibilities, and power asymmetries. The analysis attends to how geographic, institutional, and professional contexts shape their perspectives addressing RQs 1–4. First, we summarize the demographic and sectoral context of participants, then provide a thematic overview, followed by detailed analysis of four interrelated themes.

### Demographics and context

We interviewed 22 AI practitioners encompassing research/academic roles (n15), technical roles (i.e., developer/implementer/technologist; *n* = 4), and delivery/governance (i.e., project management and legal-focused work; *n* = 3). Participants worked across the public (*n* = 13), private (*n* = 5), and non-profit (*n* = 4) sectors. Geographically, they were based in West Africa (*n* = 9); Ghana accounted for 8/22 (~36%), the Caribbean (*n* = 3), South America (*n* = 3), Western Europe (*n* = 3), Southeast Asia (*n* = 2), South Asia (*n* = 1), and North America (*n* = 1; Indigenous). The sample included 13 men, 8 women, and 1 non-binary participant. Ages ranged 20 – 25 to 56 – 60 (most often 35–39). The highest education was Doctorate (*n* = 7), Master’s (*n* = 12), and Bachelor’s/Certification (*n* = 3).^2^ These distributions are not only descriptive but contextualize the transnational character of our sample

**Fig. 1:**
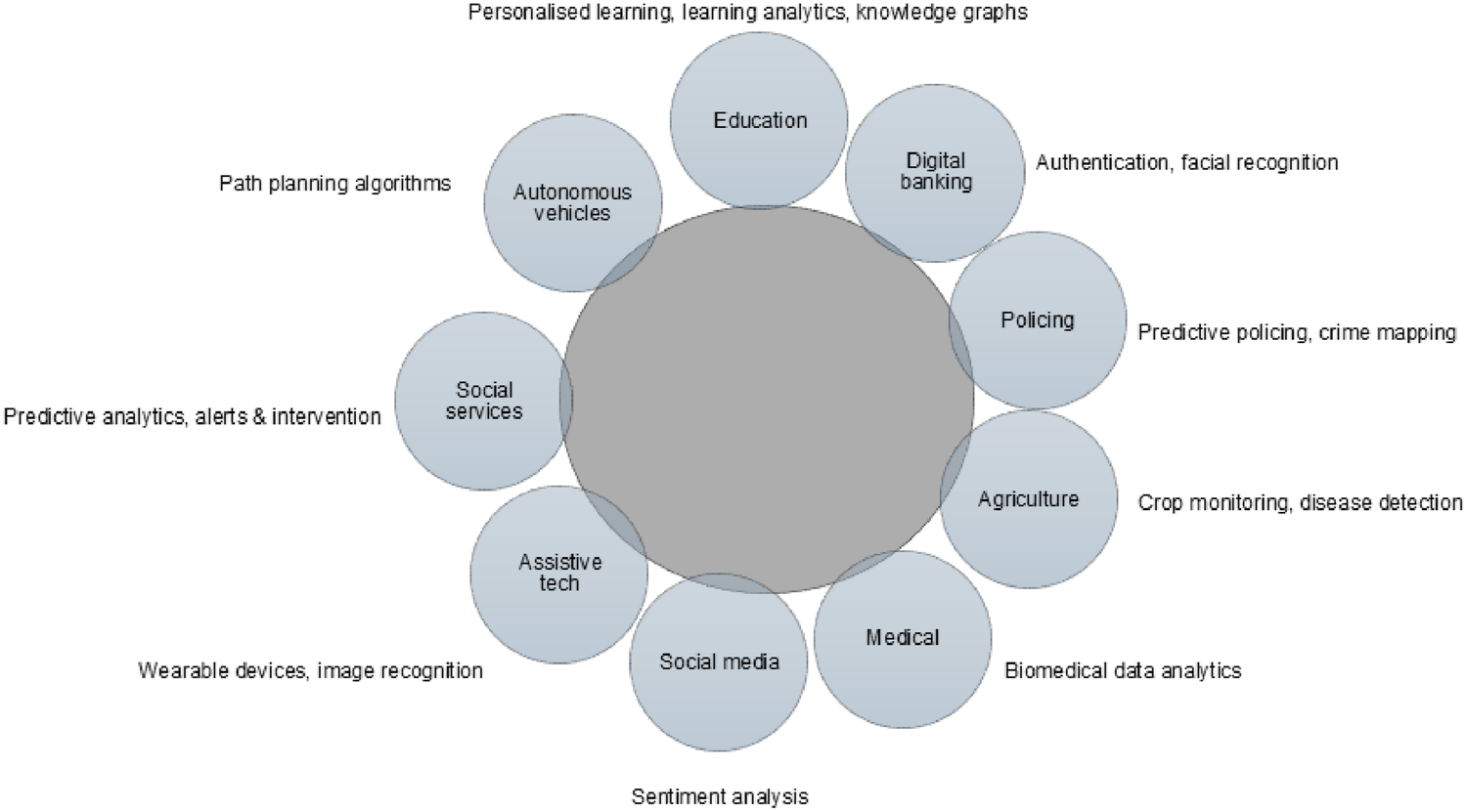
Radial layout of participants’ AI-related roles and sectors. The central node reflects shared professional engagement with AI; outer nodes show sectoral contexts and tasks

To contextualize participants’ positionalities within the global AI ecosystem, Fig. 1 visualizes their professional engagements across domains. Beyond describing diversity, the figure underscores the multi-sectoral nature of AI ethics work. Participants’ roles situate them at different points along the sociotechnical pipeline. These positions frame the interpretive contrasts explored in the subsequent themes.

The central node represents participants’ shared professional engagement with AI, while outer nodes indicate the diverse sectoral and task domains in which ethical, social, and technical questions arise. This pattern illustrates how participants occupy distinct positions within the AI ecosystem.

### Thematic overview

In analyzing the interview data, we developed four key themes. Table 2 presents the themes and sub-themes with descriptors, capturing how participants interpret AI impacts and situate themselves in relation to them. Participants’ codes indicate unique identities and contextual categories (See SI Table I).

**Table 2:** Table of themes and sub-themes

Theme	Description	Sub-themes	RQ alignment
T1. Contesting the agency of AI	Explores the belief that AI is not an autonomous system but as a human-shaped technology whose effects—incl a spectrum of harms across ethical, political, economic—reflect intent and power.	a. AI as human-shaped b. AI as the reproduction and amplification of structural inequalities	RQ1/RQ2
T2. Ethical reasoning as situated and negotiated	Examines how situational factors and constraints shape practitioners’ ethical practices.	No sub-themes reported	RQ2
T3. Navigating global power asymmetries	Explores imbalances in access, influence and decision-making framed in political-economic terms and global-local dynamics.	a. Infrastructural and material constraints b. Fragmented and unequal collaborative landscapes	RQ3
T4. Imaginaries of AI benefit and possibility	Explores the optimism about AI’s potential for growth and development and its value in local contexts.	No sub-themes reported	RQ4

### Contesting the agency of AI

This theme captures how practitioners interrogated the notion of AI’s autonomy and questioned who or what exerts influence within AI systems. Their reflections show a broader struggle over agency—between humans, institutions, and technologies—and show how understandings of responsibility and harm are negotiated.

#### AI as human-shaped

Across regions, participants described AI as a human-shaped technology rather than an autonomous entity, emphasizing that its meaning and consequences depend on the intention, values, and sociopolitical arrangements in which it is embedded. AI was frequently characterized as a malleable and empty infrastructure, one that “only functions according to the knowledge you feed it” (P01, Researcher, Europe). Participants in South America and Southeast Asia similarly emphasized AI as a tool to augment existing processes, with one practitioner noting that “It’s open and provides a solution to a problem in society. It depends on the people that use it, because it’s just a product…an algorithm that predicts and classifies” (P07, Lecturer, South America). Another practitioner explained that everything had both pros and cons which involved retaining the positive elements and discarding the negative (P05, Developer, Southeast Asia). Such accounts framed AI as logically structured, rational, and procedural, a view that often obscured its embedding within historical and sociopolitical contexts.

For participants working in education and healthcare domains, this framing also led to disciplinary silos, where AI was viewed primarily in terms of technical optimization or problem-solving. One participant described their work as “low risk” due to minimal human interaction (P03, Researcher, Southeast Asia). Another emphasized applying AI techniques to real-world issues with little thought or engagement in broader social implication (P18, Researcher, West Africa), reflecting how institutional and professional contexts can limit opportunities for deeper sociotechnical reflection. These narratives illustrate how conceptualizing AI through a deterministic lens can constrain recognition of the broader consequences and embedded values of AI tools and technologies.

This position shaped how harm was conceptualized. Rather than locating harm in AI itself, participants across Europe, Southeast Asia, and South America stressed that harm emerges from human decisions and institutional practices. Responsibility was attributed to developers, corporations, governments, and other powerful actors who act as levers of harm and “possess the money, power and the connections” to shape technological systems and their consequences (P22, Technologist, Europe). A participant from South America described this dynamic as one in which “AI enables people to cause harm in ways they always have, but now with amplified reach” (P04, Project Manager, South America). In this view, AI does not introduce new forms of harm so much as it intensifies and scales existing injustices.

However, participants also acknowledged that harms could materialize through and be legitimized by AI systems, particularly when they reproduce or amplify structural inequalities. This was especially evident among practitioners in South Asia and South America, who linked AI-driven policing, surveillance, welfare screening and commercial optimization to racialized and classed histories of state violence and economic exclusion. As one South Asian participant explained when discussing predictive policing, “AI is both reproducing and inventing historical forms of criminalisation targeting oppressed communities” (P10, Lawyer, South Asia). Practitioners from two of South America’s leading countries in AI discussed how socioeconomic disparities and heterogenous cultural dynamics influence how AI systems affect their regions.

Taken together, these perspectives challenge universalized framings of AI as either inherently beneficial or inherently harmful. Instead, they foreground AI as a sociotechnical system whose impacts are shaped by local histories, governance arrangements, and global political economies of power. This framing locates harm in the structural, institutional, and epistemic conditions that shape how AI is built and whose interest it serves and not in the technology itself.

#### Harm as the reproduction and amplification of structural inequalities

Participants’ understanding of harm followed directly from the view that AI is shaped by human and institutional decisions rather than unintended side-effects or autonomous agency. Participants conceptualized harm as structural, arising from the sociotechnical arrangements in which AI is embedded. This reflects a view of AI as entangled in histories of exploitation, racialization, data extraction, and uneven geopolitical development, whereby technologies inherit and extend the logics of the systems that produce them.

#### Job displacement and automation

Job displacement emerged as the most frequently cited and immediate form of harm. Participants across regions expressed concerns that automation threatens roles, especially in sectors characterized by routine or low-skilled tasks. AI-enabled automation was seen as reducing opportunities for stable employment and widening already fragile labor markets; one where “even coders could eventually be replaced by generative AI tools” (P05, Developer, Southeast Asia). In some context, this anxiety coexisted with cautious optimism, where AI was viewed as “increasing efficiency, but also cuts people out the equation” (P12, Researcher, Caribbean) although  potentially generating new forms of work, albeit unevenly distributed. One practitioner argued that although AI may eliminate certain jobs, it creates new ones, suggesting this shift was a challenge to be embraced (P17, Researcher, West Africa). Another contextualized automation within the broader trajectory of industrialization, noting that technological change often disrupts existing roles but ultimately drives economic growth (P04, Project Manager, South America).

Recent analysis on the impact of AI on the global labor force indicates that job displacement will likely affect high-income, AI-intensive countries, while low-income nations, especially those with limited infrastructure, will be less exposed to job losses (Demombynes et al. 2025). Medium-income countries are somewhere in between, and many of our participants are from these countries. Newly industrialized countries, like Indonesia or service economies like Antigua, Barbados, and Jamaica which do not have the same social safety nets may struggle more with transformative changes to the labor market.

#### Representational harms

Participants from South America and the Caribbean raised concerns about representational harms caused by inherent biased datasets and foundational models which led to cultural misrepresentation and erasure. Representation harms were understood as extension of colonial knowledge hierarchies where dominant cultural narratives are reproduced and legitimized through AI models. The harms shaped identity and public imagination. One participant described how AI-powered search engines often misrepresented Peruvian cultural artifacts, warning that such inaccuracies could lead to long-term misattribution and cultural erasure (P09, Researcher, South America). This is not an unfounded concern and reflects broader historical patterns, where dominant perspectives have distorted or appropriated cultural artifacts—such as the misinterpretation of the Benin Bronzes during colonial looting. Only later, when decolonial scholarship highlighted these misinterpretations and impacts of cultural appropriation, did the Western world begin to understand the consequences for knowledge that colonialism has had (Hicks 2020, p. 13).

Another participant questioned how intersecting identities, particularly race and gender, compound misrepresentation and marginalization:

*How are Black women being represented in these spaces online? And where is that information and these perspectives coming from? Who's driving that and how do we change that? Because what we generally get from the communities is that the depictions are not necessarily positive. For example, when we look at stock photos and we sometimes literally give them an activity … let's see if we can find a black female astronaut, you can't find it. It kind of demonstrates just how many inequities still exist or the prejudices that we hold in real life end up being reflected in the technology, including AI (P21,Project Manager, Caribbean).*

This was perceived as a cyclical dynamic whereby inputting culturally specific or “Black” information into AI systems that have been trained on data that reflects societal prejudices and assigns value to certain images may perpetuate harmful stereotypes. Their reflections mirror prior research showing that search algorithms often favor whiteness and discriminate against women of color (Buolamwini and Gebru 2018; Noble 2018). Brock (2020) further argues that online imagery tends to center whiteness as a universal, techno-cultural identity, raising ethical questions about increasing the visibility of Black and Brown bodies in systems and for entities that may already devalue and dehumanize them.

These concerns challenge the widely held assumption that more data will resolve representational bias. Historically, data from marginalized communities have often been extracted and used for surveillance and control, rather than empowerment. This legacy helps explain the resistance among some groups to data collection efforts, and the rise of pro-privacy and data sovereignty movements, which advocate for community control over their own data and its use in projects that serve local needs and properties.

#### Data collection, privacy, and security harms

Data collection, privacy violations, algorithmic profiling, and biometric surveillance were highlighted as pressing harms by practitioners across regions. Harms were described as continuations of existing policing and state monitoring infrastructures. In some cases, the risk was not simply data misuse, but the expansion of institutional power over marginalized communities. One participant described how data collection was reproducing and inventing historical forms of policing and criminalization of oppressed communities, such as the Hijra community—encompassing transgender and intersex individuals in India and South Asia:

*The Criminal Rights Act criminalised the Hijra communities in India for being sexually deviants. Extensive data was collected— including children—and used to justify their surveillance. Whether it’s AI or policing, the data itself becomes a self-fulfilling prophecy, reinforcing what authorities already believe (P10,Lawyer, South Asia).*

A participant with intersecting minoritized and gender-diverse identities working in a HIC expressed concerns about data misuse, stating that they were “always worried about discrimination and data leaks, which identified people and evaluated them by the data” (P22, Technologist, Europe). These fears are grounded in reality. A 2023 data leak associated with the genetic testing website 23and Me exposed genetic data of Ashkenazi Jewish and Chinese users amid rising hate incidents (DeGeurin 2024). In 2019, the HIV status of over 14,000 individuals in Singapore was leaked online (Griffiths 2019). These examples show how data collected to serve certain purposes under certain conditions can always be mobilized later if a new purpose emerges.

Participants described datafication and misinformation as harms that are deeply situated in local infrastructures and political conditions. Across West Africa, concerns focused on deepfakes and financial scams as urgent cybersecurity threats, where weak regulatory protections heighten vulnerability. A practitioner from a Muslim-majority African context explained that facial recognition systems often misclassified women who wear religious head coverings, which was a reflection of non-inclusive training datasets. In Brazil and Ecuador, participants criticized the unchecked retention and commercialization of personal data and the use of AI systems for political monitoring, noting that biometric surveillance is particularly threatening where histories of state repressions and socioeconomic inequality exist. Across Southeast Asia, Europe, and the United States, others pointed to algorithmic profiling and political polarizations in social media ecosystems, particularly around elections.

The data reveal that individuals who experience harm are often more adept at articulating the specifics of their experiences and highlighting the nuances of these harms. Having experienced the way harm happens as a process, our participants are best placed to understand (and potentially predict) systemic issues and offer valuable insights that are contextually relevant and attuned to localized conditions.

### Ethical reasoning as situated and negotiated

Our participants described ethical decision-making in AI development as largely informal, adaptive, and context-dependent, rather than embedded in structured governance processes. Among those engaged in technical work, ethical considerations were typically navigated on an ad-hoc basis, mediated by organizational culture, internal power dynamics, and resource constraints. This was the case more so than external regulation. As explained by one participant, efforts to address representational fairness, for instance, often required individual advocacy and justification, especially where such work was not seen as commercially valuable (P22, Technologist, Europe)

In some settings, fairness and accountability work was carried out by internal interest parties within organizations rather than regulated professional bodies or ethics committees. For example, a team in Brazil developed their own geographic proxies to audit facial recognition bias, underscoring how ethical practice was improvized within organizational limits rather than directed by regulation.

Participants also spoke to the tension between innovation and responsibility. One practitioner described needing to be “a responsible and irresponsible developer at the same time” (P18, Researcher, West Africa), illustrating how commitment to societal benefit can be in direct tension with the pace and priorities of technical development. Interdisciplinary engagement, particularly exposure to social science perspectives was described as slowly shifting how some technical practitioners conceptualized their obligations.

Of course, frameworks and guidance do potentially need to exist within the legal and cultural contexts where AI is happening to be able to guide conversations about the impacts of AI. In Indonesia, a developer referenced relying on the ITE law (i.e., regulation that governs electronic information and transactions) and a specialized compliance team to guide project development. In contrast, participants working in West African contexts reported a lack of regulatory frameworks and limited training infrastructures, which left individual  practitioners responsible for identifying and mitigating risks without institutional support:

*I used AI every day to build prototypes. We don't have laws or texts to regulate AI. Developers aren’t aware they’re creating biases. We need workshops and panels… I don't know, sensitization across Africa (P20, Developer, West Africa).*

Overall, these accounts illustrate that ethical reasoning was not absent, but situated and contingent, which emerged through personal initiatives, informal collaboration, and context-specific practices. Where institutional support and regulatory frameworks were limited, practitioners often shouldered the burden of ethical judgment individually. As reflected in the sample composition (see Supplementary Table S1), most West African participants worked in public-sector research roles. Their accounts, therefore, emphasized institutional hierarchies. The data reveals gaps in governance and professional training, particularly in LMIC and UMIC higher education institutions and research environments. These situated ethical practices are shaped by broader global power asymmetries in infrastructure, funding, standards and knowledge production.

### Navigating global power asymmetries

Participants highlighted how structural power imbalances inform AI development governance and deployment. We interpreted these challenges in political-economic terms in relation to nation-states, corporations, and global/local relationships. Practitioners from South America and the Caribbean shared similar critiques about the dominance of Western epistemologies and challenged the universalization of Western governance model. One practitioner challenged the assumption that Western liberal governance, its frameworks, and resources are universally applicable, arguing that such models often overlook the need for region-specific regulation:

*Political liberalism is not a preferred governance method all around the world. We’re subject to decisions made in the US or EU. It can be nice to have, like DSADMA and GDPR, but they don't address our issues; they address the European side of global issues. In Peru, we don’t have the power to regulate anything because we don't have the power as a state. For instance, all these large corporations, don’t even have offices in my country (P09, Researcher, South America) .*

This critique highlights a wider issue; the tendency to treat Western political systems as globally normativewhile framing non-Western approaches as merely regional. A Caribbean practitioner described international off-the-shelf AI solutions as ill-fitting imports that governments adopt without adequate understanding which was often driven by hype and profit rather than public benefit (P11, Researcher, Caribbean). They further mentioned that previous developments such as e-commerce and Blockchain were sold as panaceas to problems in the region and as ways of “rescuing the economy.” Collectively, these perspectives highlight that regional challenges require region-specific solutions, challenging the assumption that externally developed frameworks, whether political or technological, can be universally applied.

Participants also connected contemporary AI development to long-standing patterns of extraction, describing how data collected in African contexts is used to build systems elsewhere, without compensation, participation or control. As one practitioner stated, “most AI-powered systems and tools used on the African continent were built by people from the outside world, and all of these things are basically from our data” (P15, Lecturer, West Africa). Another expressed frustration over the lack of compensation and autonomy over data emphasizing that data is central to AI development and argued that African communities should be fairly compensated for data collected from the region (P18, Researcher, West Africa). These critiques were especially visible among Ghanian practitioners, reflecting both Ghana’s emerging role in regional AI ecosystems and the composition of our sample.

Practitioners working in HICs also mentioned the exploitative relationship between Western companies and LMICs and the attempts to shape AI while being in a situation of dependency. For instance, on reflecting on their native country Kenya, one participant argued that companies often treat GS countries as mere data exporters, overlooking local impacts and using these regions as testing grounds without meaningful accountability (P6, Researcher, Europe) Another researcher mentioned that Africa remains largely dependent on powerful countries for AI technologies, acting primarily as consumer rather than developers or decision-makers (P01, Researcher, Europe)

Yet participants were not passive in relation to these asymmetries. Across regions they described strategies for circumventing harm. These ranged from advocating for greater inclusion of local experts to the complete casting off from Western colonial power in favor of new, regionally grounded AI paradigms. Some emphasized the need to center cultural custodians and community expertise in defining ethical priorities (P14, Lecturer,  West Africa). Others emphasized political engagement (P04, Project Manager, South America; P06, Researcher, Europe) to make demands on AI governance, research-driven advocacy, and working directly with affected communities (P10, Lawyer, South Asia). Others envisioned more radical infrastructural autonomy, including African AI hubs and regionally governed compute and data infrastructures to “build our algorithms and models and adapt them to our context” (P20, Developer, West Africa). At the same time, tensions emerged. While many advocated for local sovereignty and decolonizing AI tools that would involve a process of “unlearning” to avoid replicating colonial logics (P21, Project Manager, Caribbean), others articulate for a “cosmopolitan AI approach” (P09, Researcher, South America), arguing that rather than regional or national frames, AI should ultimately serve as a global platform for shared identity and exploration.

Overall, the divergent positions also reveal tension between two ethical imaginaries: one rooted in local sovereignty and cultural specificity, and the other in global solidarity and shared norms. Both are responses to the same global power asymmetries, but they diverge in their visions of what a just AI future should look like.

#### Infrastructural and material constraints

Participants in LMIC and UMIC contexts described a lack of infrastructure and weak regulatory environments as major barriers to equitable AI development. Practitioners across West Africa (i.e., Ghana and Senegal) cited insufficient compute resources, limited datasets, and unreliable connectivity as persistent challenges. National AI strategies in parts of South America and South Asia were often perceived as symbolic rather than actionable lacking enforcement mechanisms, funding or alignment with local realities where regulatory systems were deliberately porous, enabling exploitation.

Public education and awareness were widely recognized as necessary but were deprioritized in contexts where economic precarity and daily survival supersede long-term technological planning. For West African participants, citizens lack awareness of their basic rights and have limited access to formal education (P14, Lecturer, West Africa) and were not thinking about AI but were preoccupied with “finding their next meal or finding ways to leave the country”. Participants from the Caribbean shared similar sentiments on public skepticism and resistance to technology, often rooted in past experiences with poorly implemented digital systems such as ad-hoc national ID rollouts eroding trust (P11, Lecturer, Caribbean) and the need to counter job displacement fears (P12, Researcher, Caribbean). Even in a HIC among indigenous communities, education was scarce, and there was a general misunderstanding of AI and lack of interest (P08, Technician, North America).

Our participants’ overarching concerns about a lack of education, awareness, and engagement are not unique to their respective regions. As Ali et al., (2024) point out that civil society engagement has been minimal, and grassroots ethics initiatives from individuals and communities globally have been even scarcer. The need for AI system outputs to be explainable, ensuring people understand their interactions and how decisions are made, is, therefore, a global imperative.

#### Fragmented and unequal collaborative landscapes

Across regions and institutions, participants described collaboration as uneven, extractive or symbolically consultative particularly in North–South partnerships. Power differentials surfaced through misaligned priorities, top-down decision-making and the privileging of foreign expertise over local knowledge. As one participant put it, “it’s a clash of culture … the Northern folks have some god complex feeling as if their thoughts are superior” (P18, Researcher, West Africa). This sentiment was echoed by a Caribbean participant who highlighted the misalignment between funder’s values and local contexts. For them, top-down directives often lacked flexibility and failed to account for local realities (P21, Project Manager, Caribbean). Even within regional networks, collaboration was often hindered by competition for limited funding, institutional fragmentation, and overlapping or uncoordinated initiatives, especially in AI4SG initiatives (P20, Developer, West Africa).

These reflections point to broader issues of inequities in recognition, legitimacy, and voice as to who gets to shape AI. Although regional frameworks and directives advocate for stronger cooperation, unified strategy, and knowledge sharing, how practitioners and stakeholders go about that in systematic ways remains unclear. Moreover, the pressure to collaborate with GN actors to attract investments in compute infrastructure and research and development further complicates efforts to build equitable and sustainable partnerships (Effoduh, 2024; Okolo, 2024). These dynamics also influenced how participants imagined possible AI futures

### Imaginaries of AI benefit and possibility

Despite widespread recognition of ethical risks and structural constraints, participants expressed optimism about AI’s capacity to enable social good. However, this optimism was not uniform, rather it was reflected in aspirational imaginaries of what AI *could do* if aligned with local needs, adequate resources, and supportive governance. Participants articulated these imaginaries through visions of collective uplift, economic opportunity, and everyday relief from labor, even when such benefits remained largely prospective rather than realized.

Participants across West Africa described AI as capable of addressing persistent infrastructural and social challenges, particularly via AI4SG projects in health, agriculture, and public services in which they were involved. As one practitioner stated, “the introduction of AI into the African community can solve a lot of problems” (P19, Researcher, Africa). Another framed AI as a tool of possibility, contingent on how and by whom it is implemented.

Others expressed hope that AI could reduce human effort, freeing time and attention for more meaningful work. As one participant stated, “AI does things we don’t have to do anymore” (P05, Developer, Southeast Asia). In South America, participants linked AI to educational access and informational autonomy, noting its potential to address teacher shortages (P4, Project Manager, South America) and provide alternatives in countries with gaps in civic education and fragmented media landscapes (P9, Researcher, South America).

Economic imaginaries were also prominent. Participants in Ghana, South America, and the Caribbean described AI as a growing sector capable of generating employment, attracting investment and cultivating innovation capacity. One participant described AI a potential equalizer that could allow Small Island Developing States to “catch up” and accelerate development without reproducing historical dependency:

*I believe in the age of AI… anything we're behind on; it gives us the chance to catch up. We can more effectively innovate and create software and develop our infrastructure instead of relying on existing international stuff. I think AI is greatly bridged the gap between them and us. We all have this super smart assistant that can tell us how to do things very quickly. So, it's who uses it best now instead of who has what (P12, Researcher, Caribbean).*

However, these hopeful visions often coexisted with the very harms and inequities participants described earlier. Whereas harm was narrative through lived experiences, benefits were more often framed in anticipatory, future-oriented terms which were often aligned with narratives of progress, including regional AI for development agendas and imaginaries of digital transformation. The latter is perhaps not surprising considering how ubiquitous AI technologies are influencing every aspect of daily lives Moreover, many nation states continue to capture AI as a gateway to progress and prosperity and promote sociotechnical imaginaries such as the fifth industrial revolution (Taj and Zaman 2022).

## Discussion

This study examined how AI practitioners situated in contexts across Africa, Asia, South America, and the Caribbean conceptualize AI, its societal impacts, and the ethical and geopolitical complexities surrounding its development. It also included minoritized practitioners based in Western Europe and the United States. Across these contexts, practitioners largely rejected the notion of AI as a neutral or autonomous entity, instead framing it as a human-shaped tool whose outcomes depend on the intentions, values, and power of those who design and deploy it. Yet, a dual orientation was evident. Practitioners in Europe, Southeast Asia, and parts of South America often described AI in deterministic and procedural terms, focusing on its technical components and efficiencies while bracketing broader ethical or societal implications. These accounts align with dominant GN logics that prioritize optimization and procedural governance

(Floridi and Cowls 2022; Mittelstadt et al. 2016). Such orientations, particularly prevalent among public-sector researchers and developers reflect disciplinary silos that obscure AI’s sociotechnical nature.

While some participants adopted procedural or deterministic framings, other articulated a markedly relational and sociotechnical understanding of AI. Practitioners, particularly in South Asia and parts of South America, described AI as a sociotechnical assemblage whose meaning and consequences are shaped by political economy, historical trajectories, and the uneven distribution of power and agency (Hassan 2023; Ricaurte et al. 2024).

Their accounts offer situated epistemologies that expand current understandings of AI ethics and governance beyond dominant GN frameworks. Across these narratives, harm was consistently positioned as structural, emerging from the social worlds in which AI systems are embedded. Participants described AI as exacerbating forms of exploitation already experienced through data extraction, state surveillance, and cultural marginalization. In this framing, AI does not create new injustices so much as amplify existing inequalities. These perspectives, thus, diverge from universalists and procedural approaches.

Ethical reasoning, similarly, did not appear as a stable set of principles or professional norms. Instead, participants in two West African countries described ethics as a negotiated and adaptive practice, influenced by institutional context, resource constraints, and uneven regulatory environments. In settings where formal oversight or professional guidance was weak or absent, practitioners relied on peer-based collaboration and experiential judgment to navigate ethical ambiguities. These practices reveal that ethical agency is both constrained and enacted within structural limitations. That is, practitioners operate under certain conditions yet cultivate relational forms of responsibility that respond to local realities. In doing so, their accounts challenge Eurocentric models of AI ethics that presume institutional capacity and regulatory coherence. Our findings, therefore, echo broader critiques that argue that global AI ethics frameworks embed GN assumptions of standardization and control, overlooking the context-specific practices through which ethics is produced in resource-limited and unevenly regulated environments (Alvarado 2023; Birhane 2021; Roche et al. 2021).

These adaptive practices, while locally grounded, are also symptomatic of wider structural dynamics. The very conditions that require practitioners to improvize ethically are themselves products of global power asymmetries. Participants described how these asymmetries structure the global AI ecosystem. They identified that decisions about standards, risk prioritization, and governance are largely determined by actors in the GN, while those in the GS often operate under conditions of infrastructural dependency, limited autonomy, and externally imposed technological solutions. These dynamics were understood not merely as contemporary economic disparities but as historically rooted patterns of extraction and epistemic dominance that continue to define the global AI pipeline. However, the ways power was experienced varied by regions. Participants in South America and the Caribbean emphasized the risks of adopting imported frameworks and AI solutions without contextual alignment, whereas those in West Africa foregrounded the economic and epistemic stakes of data extraction and outsourcing. Minoritized practitioners working in the GN, though relatively insulated from material harms, expressed acute awareness of exploitative relationships and the consolidation of ownership and authority. Across these accounts, participants articulated forms of agency and resistance and ways of circumventing harm through refusal, adaptation, and reimagination. In doing so, they seek to build regionally grounded AI ecosystems and alternative cultural and epistemic frameworks for evaluating technology (Bon et al. 2022; Mhlambi and Tiribelli 2023). These perspectives highlight how power not only constrains ethical possibility but also provokes new imaginaries for more equitable and contextual forms of AI governance.

Amid these enduring asymmetries, participants also articulated imaginaries of AI benefit and possibility that were aspirational, cautious and, at times, contradictory. While harms were described through lived and often immediate experiences, benefits were often framed as future-oriented potentials with visions of infrastructural improvements, economics development, and social good. However, their imaginaries were not naïve expressions of techno-optimism. Rather, they functioned as world-making gestures and assertions of agency, through which practitioners sought to shape their AI futures from within constrained material and political conditions. Participants’ visions ranged from regionally grounded and locally governed AI ecosystems to global collaborative technologies aimed at fostering shared knowledge and capacity building. They imagined futures in which AI could strengthen educational access, improve public service provisions and re-center agency within the global AI order. However, desires for epistemic and technological sovereignty also expose a tension between autonomy and assimilation. As Ali et al. (2024) caution, bottom-up or “pluriverse” ethics risk becoming little more than regional dialects within a global language of power, namely adaptation that operate within, rather than transform, dominant frameworks. Our participants’ visions of regional AI ecosystem, thus, sit uneasily between resistance and incorporation.

Across regions, the content and tone of these imaginaries diverged. Practitioners in both Ghana and Senegal positioned AI as a tool for addressing persistent infrastructural and social challenges, often through AI4SG initiatives. In the Caribbean and South America, participants imagined AI as a catalyst for investment and innovation across industries which could foster technological parity with more advance economies. These visions often aligned with GN developmental narratives echoing international and national strategies that position AI as an engine of transformation prosperity. In this regard, the circulation of imaginaries such as the Fifth Industrial Revolution (Taj and Zaman 2022) demonstrates how global discourse of digital transformation are internalized, adapted, and contested within local contexts.

Participants’ accounts show that global debates about AI cannot be adequately understood without attending to the knowledge and interpretive strategies of those working at the peripheries of global technical power. AI ethics is plural, situated, and historically entangled with global inequalities. Practitioners’ insights compel us to consider not only what AI does, but who it does things for, with, and to—and under what conditions those dynamics could be transformed.

## Conclusion

This study demonstrates that despite frequent marginalization within dominant AI discourse, practitioners’ in LMICs, UMICs and those minoritized working in HICs contexts possess expertise that is essential for understanding AI and its impacts. Their perspectives illuminate how harm materializes, how ethics is negotiated under constraint, how power operates through global infrastructures, and how futures are imagined beyond the present. By grounding decolonial AI debates in practitioners situated knowledge, this study shows how ethical agency and governance emerge under structural inequality. Meaningful progress in AI governance, therefore, requires decentring universalist frameworks, recognizing contextual expertise, and supporting locally grounded and culturally informed approaches to AI design and regulation. Without such shifts, attempts to address AI’s harms risk reproducing the very inequalities they purport to solve.

## Limitations and future research

This study’s scope and sampling inevitably shaped its insights. Recruitment through professional networks and an in-country conference led to an overrepresentation of Ghanaian participants. Rather than a flaw, this concentration reflects Ghana’s role as a regional hub in African AI ecosystems and offers a useful lens on infrastructural dependencies, data extraction, and governance capacity. As interviews were conducted primarily in English, linguistic nuance may not have been fully captured, and participants may represent a self-selecting group already engaged with ethical or governance discourse. Qualitative research of this kind is not intended to produce universally generalisable claims but to surface situated understandings and frame future inquiry. Future research should expand comparative depth across additional regions, ensure greater variability of roles and sectors, and adopt participatory designs that co-develop research with local practitioners to challenge dominant framings and explore how ethical practices evolve across diverse sociopolitical and linguistic contexts.

## Supplementary Information

Below is the link to the electronic supplementary material.

Supplementary file1 (pdf 157 kb)

Supplementary file1 (pdf 147 kb)

## Data Availability

No datasets were generated or analysed during the current study.

## Footnotes

1 https://datahelpdesk.worldbank.org/knowledgebase/articles/378834-how-does-the-world-bank-classify-countries

2 A coarsened demographic table (ID–gender–region–role–sector) using broad regional categories to protect participant anonymity is available in Supplementary Table I.
