# Supplementary material for: Understanding AI and power: situated perspectives from Global North and South practitioners
Source: AI Soc. 2025 Nov 14;41(4):3981–96. doi: 10.1007/s00146-025-02731-x (PMC13124877; doi:10.1007/s00146-025-02731-x)
Supplement: Supplementary file 1 — Supplementary file1 (pdf 157 kb) [file 146_2025_2731_MOESM1_ESM.pdf]

Supplementary Information for: "Understanding AI and Power: Situated Perspectives from Global North and Global South Practitioners"

Journal: AI & Society

Authors: V. Brown, R. Larasati, J. Kwarteng, T. Farrell. Corresponding author: venetia.brown@open.ac.uk

Supplementary Table I. Coarsened participant demographics used for quotation tags.

ID-gender-region-role-sector mapping of study participants. All attributes are aggregated into broad categories to reduce deductive disclosure.

**ID**	**Gender**	**Nationality**	**Region**	**Education**	**Profession**	**Role family**	**Sector**
P01	Man	Nigerian	Western Europe	Doctorate	Researcher	Research/academic	Public
P02	Woman	Ghanian	West Africa	Masters	Lecturer	Research/academic	Public
P03	Woman	Indonesian	Southeast Asia	Masters	Researcher	Research/academic	Public
P04	Man	Brazilian	South America	Masters	Project Manager	Delivery/governance	Private
P05	Man	Indonesian	Southeast Asia	Masters	Developer	Technical	Private
P06	Woman	Kenyan	Western Europe	Masters	Researcher	Research/academic	Public
P07	Man	Ecuadorian	South America	Masters	Lecturer	Research/academic	Private
P08	Man	Indigenous American	North America	Bachelors	Technician	Technical	Private
P09	Man	Peruvian	South America	Doctorate	Researcher	Research/academic	Private
P10	Woman	Indian	South Asia	Masters	Lawyer	Delivery/governance	Non-profit
P11	Man	Barbadian	Caribbean	Doctorate	Lecturer	Research/academic	Public
P12	Man	Antiguan	Caribbean	Undergrad	Researcher	Research/academic	Public
P13	Woman	Ghanian	West Africa	Masters	Lecturer	Research/academic	Public
P14	Man	Ghanian	West Africa	Doctorate	Lecturer	Research/academic	Public
P15	Man	Ghanian	West Africa	Masters	Lecturer	Research/academic	Public

P16	Man	Ghanian	West Africa	Masters	Researcher	Research/academic	Public
P17	Man	Ghanian	West Africa	Doctorate	Researcher	Research/academic	Public
P18	Man	Ghanian	West Africa	Doctorate	Researcher	Research/academic	Public
P19	Woman	Ghanian	West Africa	Doctorate	Researcher	Research/academic	Public
P20	Woman	Senegalese	West Africa	Masters	Developer	Technical	Non-profit
P21	Woman	Jamaican	Caribbean	Masters	Project Manager	Delivery/governance	Non-profit
P22	Non-binary	Egyptian	Western Europe	Certificate	Technologist	Technical	Non-profit
