# Supplementary material for: Understanding AI and power: situated perspectives from Global North and South practitioners
Source: AI Soc. 2025 Nov 14;41(4):3981–96. doi: 10.1007/s00146-025-02731-x (PMC13124877; doi:10.1007/s00146-025-02731-x)
Supplement: Supplementary file 2 — Supplementary file1 (pdf 147 kb) [file 146_2025_2731_MOESM2_ESM.pdf]

Supplementary Information for: “Understanding AI and Power: Situated Perspectives from Global North and Global South Practitioners”

Journal: AI & Society

Authors: V. Brown, R. Larasati, J. Kwarteng, T. Farrell. Corresponding author: venetia.brown@open.ac.uk

Supplementary Table II. Codebook including themes, subthemes, definitions and quotations from the dataset.

**Themes**	**Subthemes**	**Definitions**	**Examples from Transcripts**
Contesting the Agency of AI	AI as Human-Shaped	AI conceptualised as a human-shaped technology rather than an autonomous system, with its effects understood as reflections of human intent and power	“Literally the power is in our hands and it’s what we do with it. Whether we use that responsibly or not is what determines the outcomes that we get” (P21-PMgr-Caribbean)<br>“It’s never about the technology. It’s about who has control over that technology and when I started working in tech, I started seeing catastrophic people who are like problematic and possess the money, power and the connections, or like, abusing people to develop technology” (P22-Tech-Europe)
	AI as the Reproduction and Amplification of Structural Inequalities	AI understood as producing harms that extend across ethical, political, and economic spheres, reveals concerns about inequities, abuse of power and value distributions	“I think there is reasonable concern about biometrics, facial recognition etcetera, because it can be used by repressive governments and police forces” (P04-PgrM-S. America)<br>“One example is the Cambridge Analytica scandal when it happened. Also, social media accounts in Africa and other Global South countries were affected with misinformation but those were not really taken into account as much as when it happened later in the UK with Brexit” (P06-Rschr-Europe)
Ethical Reasoning as Situated and Negotiated	No subthemes reported	Ethical decision making characterised as fluid and situational, reflecting participants’ adaptive response to contextual uncertainty	“I didn’t receive any instructions and that’s why I mentioned the [named summit] in Europe saved me. I did whatever I wanted in the past because I just built solutions, but I didn’t have the ethical skills. I didn’t know what the definition of responsible AI was” (P20-Dev-W. Africa)<br>“Within the lab, we have someone…our policewoman who checks is this possible, is that possible. It’s annoying but vital to the development. Once you’ve answered all her questions and added the development it would benefit society. We’ve grown to love questions. She’s more or less our compliance officer” (P18-Rschr-W. Africa)
Navigating Global Power Asymmetries	Infrastructural and material constraints	Limitations such as inadequate infrastructure, weak regulation, insufficient compute resources and lack of enforcement mechanisms viewed as barriers	“I know that there are laws in the EU around fairness and speaking to ethics in AI. In India, the legal and regulatory frameworks around this are extremely porous and are designed to do this” (P10-Lwyr-S Asia)<br>“The main challenges is infrastructure. Since 2017, I heard about some data centres in Senegal. We are in the field and world of AI and until now the data centres are not operational” (P20-Dev-W. Africa)
	Fragmented and unequal collaborative landscapes	Collaboration seen an unequal reflecting disparities in resources and visibility between GN/GS stakeholders	“We have expertise. We have knowledge, but still they will bring somebody from overseas who come say. Okay, what’s the problem and what do you think. They would type that in a report and present it to whoever brought them here. They come with a prefabricated solution that doesn’t quite fit the puzzle” (P11-Lect-Caribbean)
Imaginaries of AI Benefit and Possibility	No subthemes reported	Participants beliefs about AI’s potential for growth and development and its value in local contexts	“We are being more productive with less resources, that’s the thing and everything is thanks to the AI tools that we’re using in our teaching. It will be easier to get the Metaverse so maybe this will be better for education. I believe in terms of AI evolution we will get more solutions to reduce the gap” (P07-Lect-S. America)
